# Analysis of the dynamic changes in gut microbiota in patients with different severity in sepsis

**DOI:** 10.1186/s12879-023-08608-y

**Published:** 2023-09-19

**Authors:** Yanli Liu, Yanan Guo, Su Hu, Yujun Wang, Lijuan Zhang, Li Yu, Feng Geng

**Affiliations:** Intensive Care Unit, The Central Hospital of Wuhan, Tongji Medical College, Huazhong University of Science and Technology, Wuhan, China

**Keywords:** Sepsis, Gut microbiota, Dynamic changes, 16S rRNA gene sequencing, Intensive care unit

## Abstract

**Background:**

The gastrointestinal tract contains a massive microbiota, and targeting the gut could be a potential intervention for sepsis. However, the interaction between sepsis and the intestinal microbiota is defined as an “incompletely understood bidirectional relationship”.

**Methods:**

This retrospective observational cohort study investigated the fecal microbiota of sepsis patients admitted to the Department of Critical Care Medicine of the Central Hospital of Wuhan, China, from May 2019 to January 2020. 14 septic patients were divided into the non-severe group and the severe group according to the Acute Physiology and Chronic Health Evaluation II (APACHE II) score. Herein, fecal samples were serially collected on admission, the third, fourth, and fifth days, and ICU discharge. The fecal microbiota was analyzed by 16S rRNA gene sequencing and its correlation with clinical parameters was evaluated.

**Results:**

Bacteroidetes, Firmicutes, and Proteobacteria were dominant phyla at ICU admission, and fecal biodiversity was not significantly different between the non-severe group (APACHE II < 15) and the severe group (APACHE II > 15). However, the diversity of the gut microbiota was significantly lower at ICU discharge than that at ICU admission with the extension of treatment time. Further significant difference flora analysis (LEfSe) showed that the genera *Veillonella* and *Ruminococcus* were the most discriminant biomarkers at ICU admission in non-severe and severe patients, respectively, while *Enterococcus* was the most discriminant biomarker at ICU discharge in all septic patients. Of note, liver function tests, including ALT, AST, TBIL, and DBIL correlated with the prevalence of various bacterial genera.

**Conclusions:**

The diversity of the gut microbiota in patients with sepsis decreases dramatically during ICU stay, and there are distinct dynamic changes in gut microbiota among patients with different severity in sepsis.

**Supplementary Information:**

The online version contains supplementary material available at 10.1186/s12879-023-08608-y.

## Background

Sepsis is a life-threatening organ dysfunction caused by the host's maladjusted response to infection. If the infection is not well controlled, it can develop into septic shock or even multiple organ dysfunction syndrome (MODS) [1]. More than 48.9 million people worldwide develop sepsis annually, and nearly 11 million of them die, thereby leading to the World Health Organization recognizing sepsis as a global health priority [2, 3]. However, although the definition of sepsis has evolved in recent decades, the treatment approach including prompt use of appropriate antibiotics with supportive measures and source control has remained much the same [4]. In recent years, with the emergence of the microbiome, metabolome, and molecular epidemiology, accumulating evidence suggests that the gut may be a potential intervention target for sepsis and MODS [5, 6].

In addition, the gastrointestinal tract contains a massive and diverse number of microorganisms that compose a complex ecosystem known as the gut microbiota that fulfills various functions including digesting nutrients, stimulating immune system development, and protecting against potential pathogens by competition [7]. The interaction between sepsis and the intestinal microbiota is defined as an “incompletely understood bidirectional relationship”. On the one hand, microbiota disruption leads to increased susceptibility to sepsis [8]. On the other hand, the intestinal microbiota composition is affected by sepsis and its treatment (particularly broad-spectrum antibiotic use) [5, 6]. Therefore, an understanding of sepsis-related gut microbiota is of great significance. Despite the increased recognition of the effect of intestinal microbiota on sepsis [9], there were few studies on its dynamic changes in patients with sepsis.

In this study, we detected the composition of the gut microbiota at multiple time points in stool samples from ICU septic patients using 16S rRNA gene sequencing, aiming to characterize the transformation of the gut microbiota in patients with sepsis and its correlation with clinical parameters.

## Methods

### Subjects

This retrospective observational study was carried out with ICU patients admitted to the Central Hospital of Wuhan, China, from May 2019 to January 2020. This study was approved by the ethics committees of the Central Hospital of Wuhan (Ethics approval NO: 2021.19). All patients were at least 18 years old and met the sepsis criterion. The diagnostic criteria of sepsis were adopted based on The Third International Consensus Definitions for Sepsis and Septic Shock [1]. However, patients who had taken antibiotics within 3 months before admission or had an expected ICU stay of < 24 h were excluded. Patients undergoing ileostomy or colostomy or with terminal illnesses were also excluded.

### Data collection and sampling

Herein, case report forms, nursing records, and laboratory findings were reviewed. Information on demographic data, underlying comorbidities, and laboratory results were also recorded on admission. The Acute Physiology and Chronic Health Evaluation II (APACHE II) score were recorded within 24 h after admission, and the Sequential Organ Failure Assessment (SOFA) score was recorded daily. Additional variables, such as antibiotic administration, the site of infection, and outcome variables (ICU stays and discharge condition), were also recorded. All fecal samples were obtained by nurses. Meanwhile, based on the APACHE II score [10, 11], patients with sepsis were divided into the non-severe group (APACHE II < 15) and the severe group (APACHE II > 15). Herein, the first sampling of the above two groups at ICU admission upon inclusion criteria verification were defined as A and B, respectively. Then, after admission, other stool samples were collected serially from patients. The A1/B1 samples consisted of peri-rectal swabs obtained in post admission day 3, 4, and 5, whereas the A2/B2 samples consisted of peri-rectal swabs collected at ICU discharge. Meanwhile, if patients produced more than one stool sample on the same day, only one was collected for testing. However, if patients were unable to produce a stool specimen on any day of the observation, defecation was performed by nurses using a glycerin enema. Fresh stool samples were stored at − 80 °C until further processing.

### Fecal microbiota analysis

Fecal samples were then sent to NOVOGENE Company Limited (Beijing, China). Herein, fecal samples were sequenced for the V3/V4 hypervariable regions of the 16S rRNA gene using the Illumina MiSeq platform. Each sample data was separated from the off-machine data based on the barcode and PCR amplification primer sequences. After the barcode and primer sequences were cut off, FLASH was used to splicer reads of each sample, and the splicing sequence was the original Tags data. Then, the fastP software was used to get high-quality Tags data through strict filtering of Raw Tags obtained by splicing. Afterward, Tags obtained after the above processing need to be processed to remove the chimeric sequence. Herein, the Tags sequence was compared with the species annotation database to detect the chimeric sequence and finally removed and the chimeric sequence was to obtain the final effective data. In addition, the Uparse algorithm was used to cluster all effective Tags of all samples, and operational taxonomic units (OTUs) were clustered at 97% sequence similarity by default [12–15].

Hence, we divided the cohort into two groups based on the APACHE II score to investigate whether microbiota diversity was associated with disease severity [10, 11]. Herein, the alpha- and beta-diversity were calculated by Qiime software. The Shannon index and Simpson index were selected to measure fecal microbial diversity. Meanwhile, the significance of dissimilarity was calculated by Principal Coordinate Analysis (PCoA) and similarity analysis (anosim) tests. Moreover, the linear discriminant analysis effect size (LEfSe) was used to analyze the bacterial community dominance between groups. Spearman's correlation analysis was used to evaluate the relationship between related parameters.

### Statistical analysis

The clinical data were performed using R (https://www.R-project.org, version 3.5.2). Continuous variables are presented as median (IQR), and Categorical variables are presented as frequencies and percentages. Furthermore, a two-sided *P*-value of < 0.05 was considered statistically significant. The graphs were created using Graphpad Prism (Graphpad Software, San Diego, CA).

## Results

### Characteristics

Fourteen patients with sepsis were included in the study based on the inclusion and exclusion criteria. A total of 40 fecal samples were collected from these patients (median age 60 years; 64% male) after ICU admission. Table 1 describes the characteristics of sepsis patients stratified by APACHE II score.

**Table 1:** Comparison of demographic characteristics between two groups of patients with sepsis

Variable	APACHE II score < 15	APACHE II score > 15	*P* value
***N***	7	7	
**Demographics**			
Age	60[53–73]	66[41–72]	0.92
Male	4(57.1%)	5(71.4%)	0.577
BMI	26[21–29]	22[17–25]	0.02
**Chronic comorbidity**			
Hypertension	2(28.6%)	3(42.9%)	0.577
Diabetes	3(42.9%)	2(28.6%)	0.577
Cardiopathy	0	1(14.3%)	0.299
**Severity of disease on ICU admission**			
SOFA score	8[2–11]	12[7–23]	0.04
Mechanical ventilation	1(14.3%)	4(57.1%)	0.094
Need of vasopressors > 1 day	4(57.1%)	6(85.7%)	0.237
**Primary infection site**			
Urinary tract infection	3(42.9%)	3(42.9%)	1
Biliary tract infection	1(14.3%)	2(28.6%)	0.515
Bowel perforation	2(28.6%)	1(14.3%)	0.515
Pulmonary infection	0	1(14.3%)	0.299
Ovarian abscess	1(14.3%)	0	0.299
**Need of antibiotics > 2 classes**	0	3(42.9%)	0.051
**Length of ICU**	3[2–7]	8[3–20]	0.069
**Death**	0	2(28.6%)	0.127

Data are presented as number (percentage) for categorical data and median (interquartile range) for continuous data

### Microbiome features upon admission

The taxonomic composition of the gut microbiota using 16S rRNA gene analysis in non-severe and severe sepsis patients at the phylum level at initial sampling (A/B) at ICU admission as determined is shown in Supplemental Figure 1. Bacteroidetes, Firmicutes, and Proteobacteria were the predominant phylum in most patients (Supplemental Figure 1). Herein, alpha- and beta-diversity methods were used to evaluate the microbial diversity and microbial community structure of intestinal microbiota samples. Then, the alpha diversity was measured using Shannon and Simpson. However, there was no difference observed in fecal biodiversity between A and B (*P* > 0.05). Then, the beta diversity analysis was performed using PCoA and anosim. Neither PCoA nor anosim differed between A and B (*P* > 0.05).

**Fig. 1:**
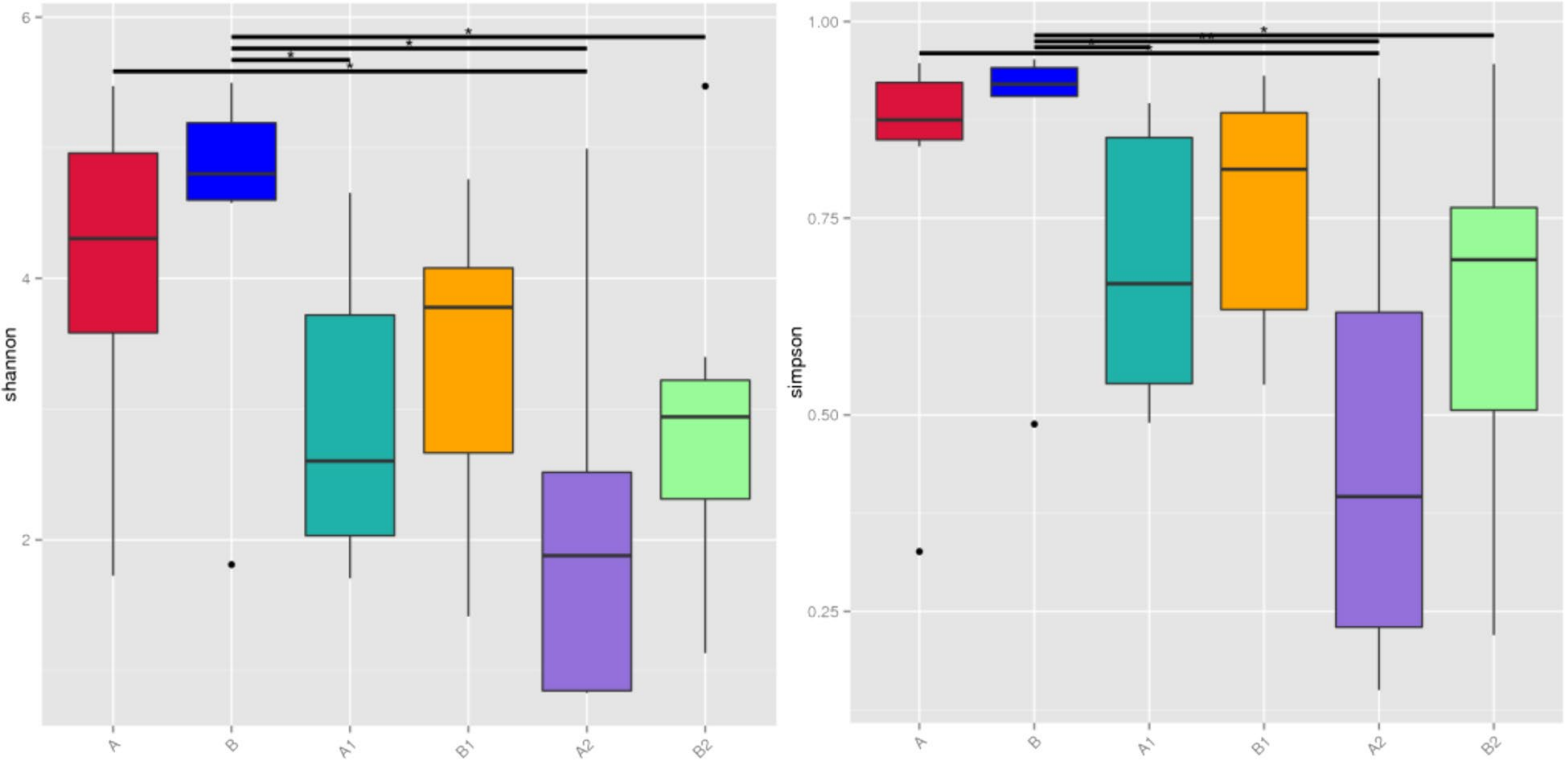
α-diversity indexes of the fecal microbiota in sepsis patients over time. A/B: The first sampling of the non-severe and the severe sepsis patients at ICU admission, respectively. A1/B1: Fecal samples in post admission day 3, 4, and 5. A2/B2: Fecal samples in non-severe and severe sepsis patients at ICU discharge, respectively

### Short-term microbiota evolution during ICU stays

**Fig. 2:**
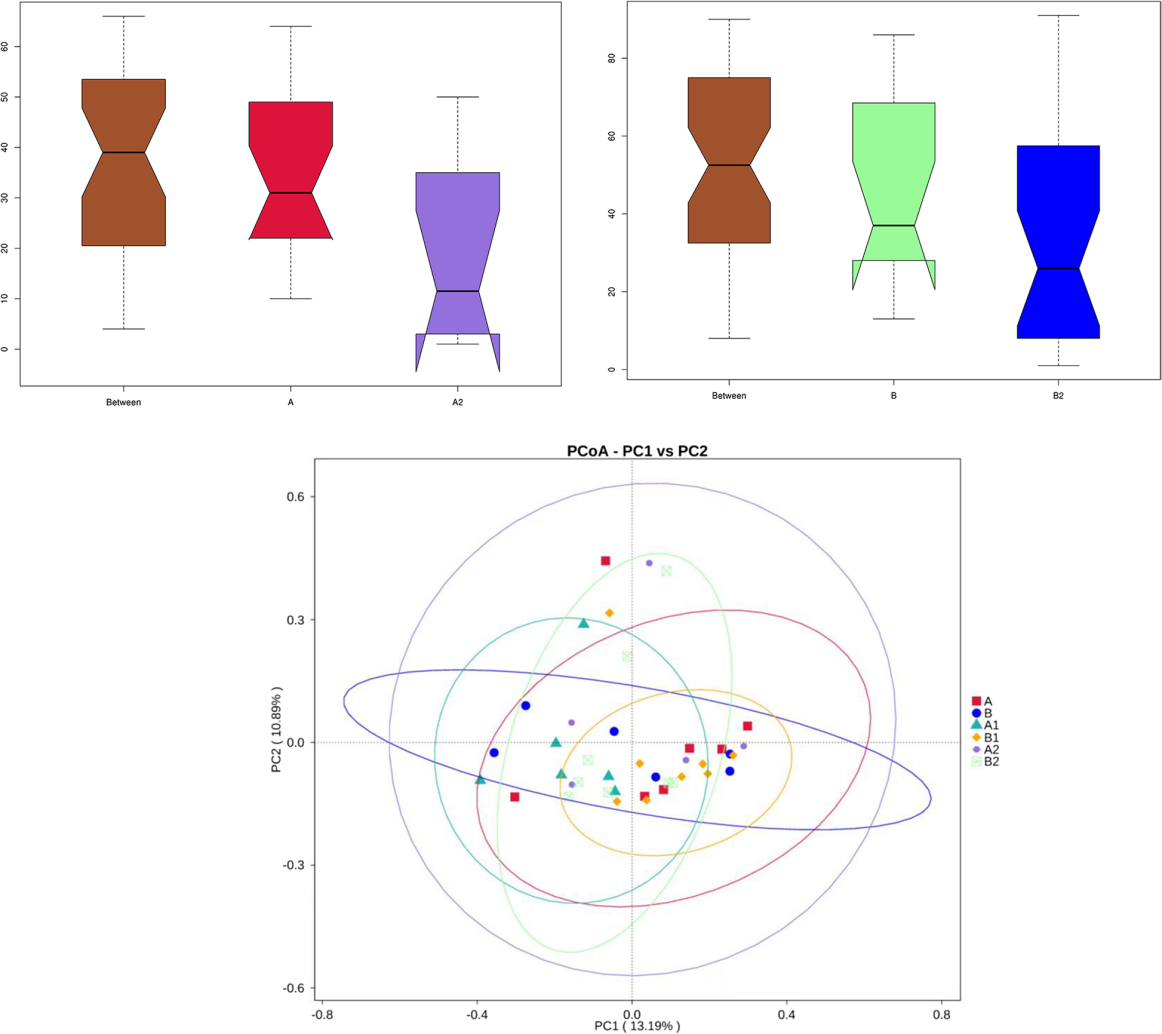
β-diversity indexes of the fecal microbiota in sepsis patients over time. The ovals reflect the clustering of the fecal microbiota. A/B: The first sampling of the non-severe and the severe sepsis patients at ICU admission, respectively. A1/B1: Fecal samples in post admission day 3, 4, and 5. A2/B2: Fecal samples in non-severe and severe sepsis patients at ICU discharge, respectively

**Fig. 3:**
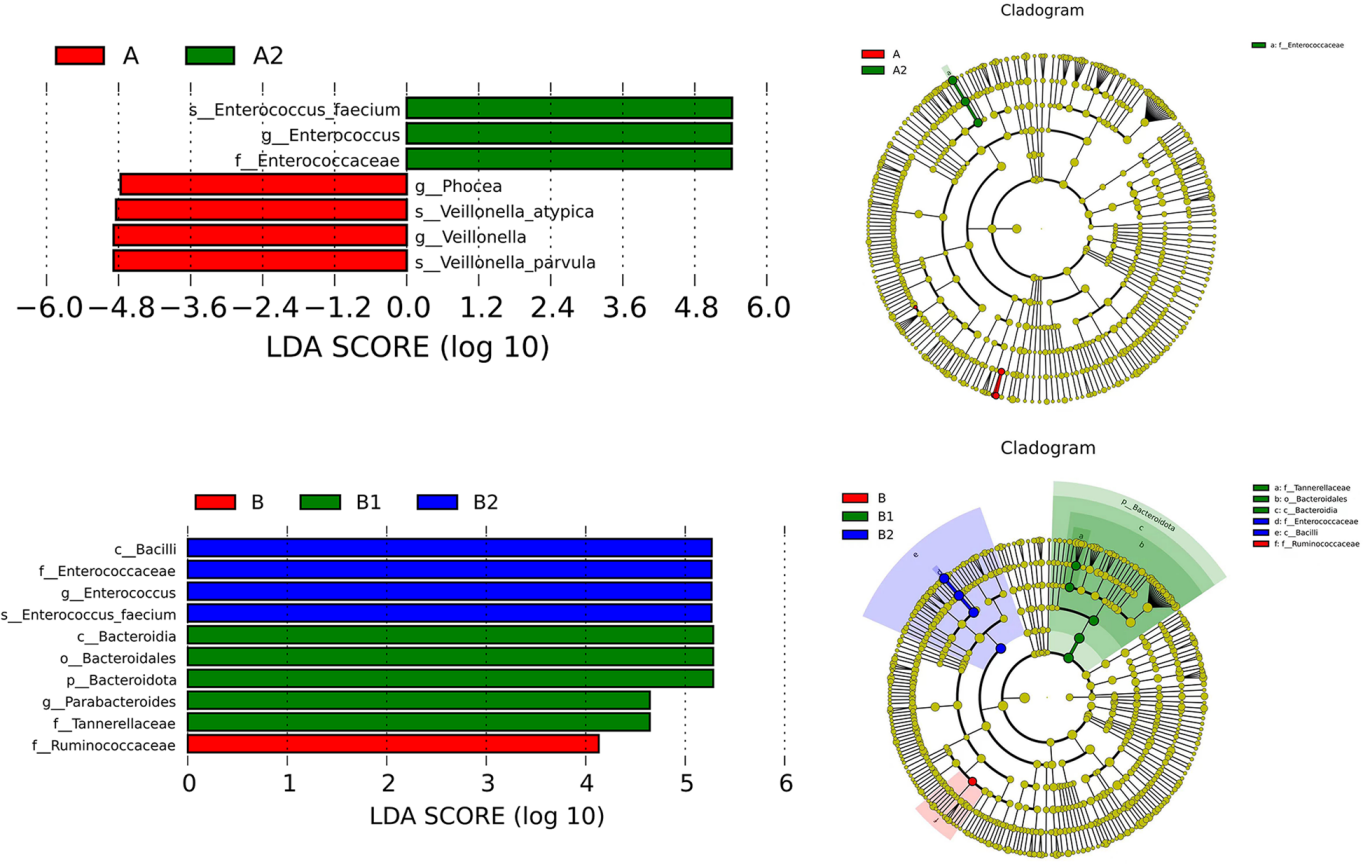
Linear discriminant analysis scores indicating differences in the bacterial taxa in patients with sepsis in each group over time. For the non-severe sepsis patients, A and A2 represented fecal samples at ICU admission and ICU discharge, respectively. For the severe sepsis patients, B and B2 represented fecal samples at ICU admission and ICU discharge, respectively

Herein, a total of 40 longitudinal samples were evaluated to discern potential changes in the diversity of samples across the ICU stay. The composition of gut microbiota was evaluated by the ratio of Firmicutes to Bacteroidetes (F/B). There was no difference observed in the ratio of F/B between the non-severe (A) and the severe sepsis patients (B) at ICU admission. However, by dynamic evaluation of the F/B ratio, we found that two out of seven patients had extreme values of F/B ratio in non-severe (A2) and severe sepsis patients (B2) at ICU discharge, respectively (*P* = 0.0761, *P* = 0.1641) (Supplemental Figure 2). Then, for all patients with sepsis, the alpha diversity assessment showed that the diversity of A2/B2 was significantly lower than that of A/B (Fig. 1). Beta-diversity, measured as anosim, significantly changed at B2 as compared to B (*P* < 0.05). Meanwhile, PCoA was used to determine the clustering pattern of microbial composition. During admission, the composition of the gut microbiota shifted from baseline to away from the original profile (Fig. 2). Moreover, LEfSe analysis was used to verify significant differences between A/B and A2/B2. The genera *Veillonella* and *Enterococcus* and the genera *Ruminococcus* and *Enterococcus* were the most discriminant biomarkers at A/A2 and B/B2, respectively. With the extension of treatment time, *Enterococcus faecium* gradually becomes the dominant bacteria (Fig. 3).

### Antibiotics

During admission, all patients were treated with systemic antibiotic therapy containing carbapenems, third-generation cephalosporins, and other antimicrobial agents, ranging from one to three (Supplemental Figure 3). Herein, carbapenems are the most commonly used antibiotics. The number of antibiotics used in patients with APACHE II score > 15 was significantly higher than that in patients with APACHE II score < 15 (*P* < 0.01), but there was no difference found in microbiota composition at the phylum level between the two groups.

### Microbiota composition in relation to clinical parameters

**Fig. 4:**
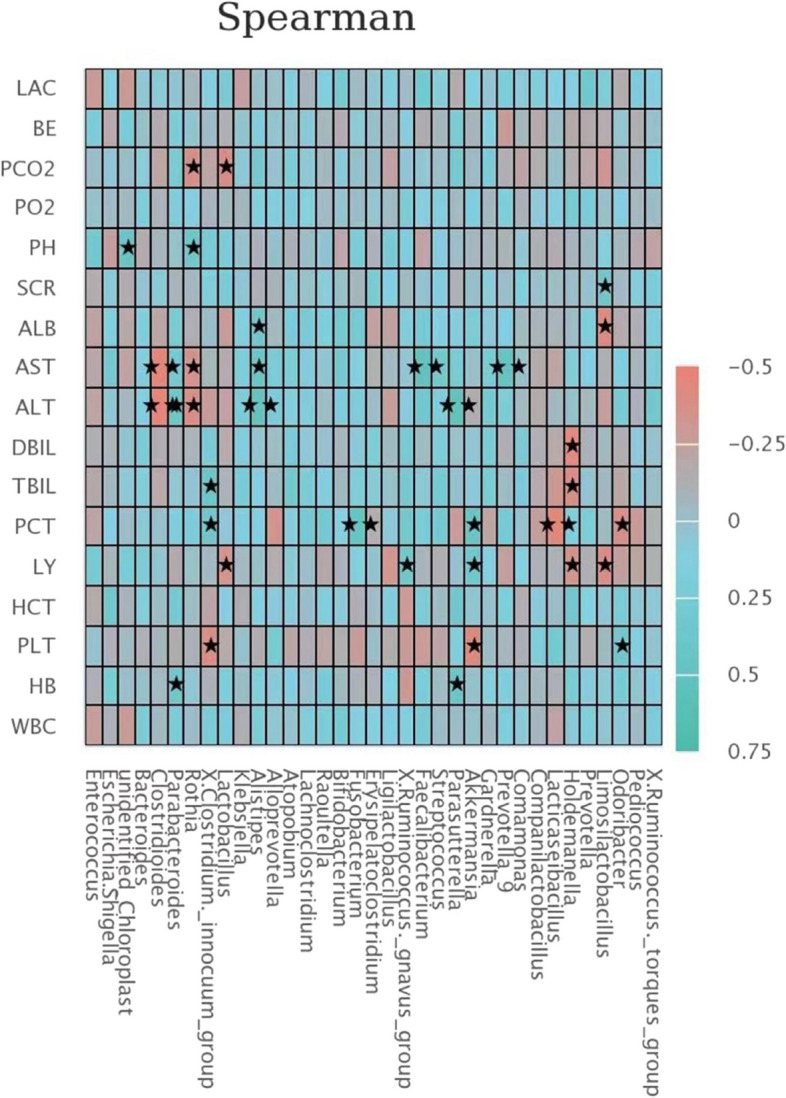
Correlation between microbiota composition and clinical parameters in patients with sepsis

Organ dysfunction is common in patients with sepsis. Thus, Spearman's correlation analysis was further used to evaluate the potential relationship between gut microbiota and clinical parameters in sepsis. As shown in Fig. 4, abnormal liver function was the most evident. *Clostridioides*, *Rothia*, and *Holdemanella* were negatively associated with liver function tests, including ALT, AST, TBIL, and DBIL. Conversely, *Parabacteroides*, *Alistipes*, *Faecalibacterium*, *Parasutterella*, and *Prevotella* were positively associated with ALT and AST. In addition, our study showed the relationship between the abundance of gut microbiota components and PCT, which reflects the active level of the systemic inflammatory response. Moreover, *Lacticaseibacillus* and *Odoribacter* were negatively correlated with PCT, while *Fusobacterium*, *Akkermansia*, and *X. corstridium* were positively correlated with PCT.

## Discussion

The intestinal tract, as the largest bacterial reservoir of the body, is regarded as the engine of sepsis and an important source of infection [16]. Understanding changes in the gut microbiota in sepsis patients is an important goal. Although the majority of current studies revealed that a large interindividual variation in intestinal microbiota dysregulation and low bacterial diversity was observed in critically ill patients [17, 18], no research has investigated the dynamic changes of the gut microbiota and its relationship with organ function in sepsis. In the present study, patients with sepsis were divided into the non-severe group and the severe group, according to the APACHE II score. the alpha- and beta-diversity at ICU admission were compared between the above two groups, and the dynamic changes of the gut microbiota before and after treatment were evaluated. Our study highlights the following: (a) the diversity of the gut microbiota between the non-severe and the severe sepsis patients at ICU admission did not change significantly; (b) with the extension of treatment time, not only the severe group but also the non-severe group had a significantly lower diversity of the gut microbiota at ICU discharge than that at ICU admission, leading to the expansion of clinically familiar pathogenic bacteria *Enterococcus faecium*; and (c) abnormal liver function correlated with the prevalence of various bacterial genera.

The gut microbiota is mainly composed of Bacteroidetes, Firmicutes, Actinobacteria, and Proteobacteria, of which Bacteroidetes and Firmicutes account for more than 90%. Herein, Bacteroidetes are Gram-negative bacteria that digest complex polysaccharides by releasing volatile short-chain fatty acids which regulate the growth of intestinal epithelial cells. Meanwhile, Firmicutes are Gram-positive bacteria that promote intestinal epithelial health and induce colonic T-regulatory cells by releasing butyrate. The main characteristics of intestinal microbiota in critically ill patients have decreased the beneficial commensal bacteria and increased pathogenic bacteria [19]. A retrospective cohort study of 10,996 hospitalized patients found a dose–response relationship between the degree of intestinal microecological disturbance and the subsequent development of severe sepsis [20]. Moreover, a prospective observational study of 24 ICU patients documented a dramatic absence of gut microbial diversity and pathogen domination of the gut microbiota in a high proportion of critically ill patients [21]. Unlike most previous studies that described the characteristics of the gut microbiota on admission, we mainly described the dynamic changes of the gut microbiota in patients with sepsis during treatment. Although, fecal biodiversity was not significantly different between the non-severe and the severe sepsis patients at ICU admission, the diversity of the gut microbiota in both patients with severe and non-severe sepsis was significantly lower at ICU discharge than that at ICU admission, potentially leading to the enrichment of the pathogenic bacteria *Enterococcus faecium*. The gut microbiota is thought to influence sepsis by bacterial translocation and preventing multidrug-resistant bacteria colonization [22, 23]. Whether the intestinal bacteria colonizes or becomes pathogenic in critically ill patients depends on the intestinal environment [24]. A study showed that the “normal” microbiota exposed to opioids can be replaced by an ultra-low diversity community of resistant pathogens in ICU patients with prolonged stays [25]. Sepsis-associated mortality is mainly attributed to subsequent secondary infections. Therefore, this finding reminds us to focus on the dynamic changes in gut microbiota in critically ill patients.

In critically ill patients, shock, inflammation, impaired immune function, drugs, change in nutrition patterns, and other factors could contribute to changes in gut microbiota [24, 26]. The main treatment for sepsis is the use of broad-spectrum antibiotics. Antibiotics are known to cause a loss of diversity in the gut microbiota and promote the growth of resistant microorganisms. Therefore, it is not surprising that patients with sepsis have intestinal microecological disorders. Intravenous administration of the broad-spectrum antimicrobial agent meropenem, other than other antibiotics, including piperacillin/tazobactam, was significantly associated with loss of gut microbial diversity, which led to nosocomial transmission of the pathogen *Enterococcus faecium* [21]. Most patients received carbapenems treatment in our study. Herein, we observed a significant increase in the abundance of *Enterococcus faecium*, which belongs to *Lactobacillus* in Firmicutes. *Enterococcus* is considered a symbiotic organism of the human gastrointestinal tract. *Enterococcus faecalis* and *Enterococcus faecium* are the most abundant *Enterococcus* species in the human gut microbiota. Once ectopic, they can cause respiratory tract infection, urinary tract infection, abdominal infection and sepsis. *Enterococci*, particularly *Enterococcus faecalis* and *Enterococcus faecium*, are important causes of secondary infections and have become a major issue worldwide. Under the pressure of using various broad-spectrum antibiotics, *Enterococci* have developed resistance to β-lactam, vancomycin, and other drugs, which undoubtedly increases the difficulty of treatment. therefore, virulence factors of *Enterococci* have been extensively studied. Unlike *Streptococci* and *Staphylococci*, most *Enterococci* do not produce proinflammatory toxins, which mediate adhesion to host tissues through genes encoding adhesion proteins. In disease states, adhesion and biofilm formation are important characteristics of bacterial ectopia in the colon lumen, and *Enterococci* adhere to host tissues through adhesion matrix molecules of their surface components and then cause infection [27, 28]. Acm (collagen-binding protein) is an adhesion protein produced by *Enterococcus faecium*, and it can bind collagen in host tissues. In addition, Acm deletion could inhibit collagen adhesion of *Enterococcus faecium* and attenuate the pathogenicity of *Enterococcus faecium* in animal models of infective endocarditis [29]. Cell wall-associated enterococcal surface proteins contribute to cell adhesion and biofilm formation of *Enterococcus faecium*, which play an important role in the pathogenesis of urinary tract infection and infective endocarditis [28]. A prospective, observational case–control study concluded that the pathogens of secondary infection in patients with sepsis might originate from the intestinal colonization of pathogens after broad-spectrum antibiotic treatment [6]. Thus, high-throughput sequencing of gut microbiota may identify patients at high risk of secondary infections in sepsis.

Moreover, intestinal microbiota plays a vital role in maintaining the stability of the intestinal environment, and its imbalance promotes liver damage. Under normal circumstances, intestinal mucosa allows a small number of LPS to pass into the portal vein. In contrast, the imbalance of gut microbiota, impaired intestinal mucosal barrier function, and increased permeability of the cell bypass barrier could lead to a large number of LPS entering the circulatory system. Herein, LPS induces intestinal tissue ischemia and hypoxia, exacerbates intestinal mucosal barrier damage, and causes bile acid metabolism disorder, and insulin resistance, thereby leading to liver damage by activating inflammatory factors [30–32]. Interestingly, our study showed that abnormal liver function is associated with dysbiosis. Hence, future studies will be required to confirm the potential of promoting recovery of liver function through improving intestinal function in septic critically ill patients.

### Study limitations

This study is subjected to several limitations. Firstly, the sample size of this group is limited, and the heterogeneity of critically ill patients is considerable. Thus, more extensive and multicenter studies are necessary to support these findings. Secondly, antibiotic therapy is necessary in sepsis, and the effect of antibiotics on intestinal microflora cannot be ignored. Animal experiments will be conducted to further verify the role of intestinal microflora in sepsis. Thirdly, although our study showed short-term microbiota disruption in patients with sepsis, we lacked follow-up studies to determine whether the presence of opportunistic pathogens led to later secondary infections and thus prolonged hospital stay. Fourthly, the causality could not be established because of the nature of the observational study and further prospective intervention study is needed.

## Conclusion

A large intra-individual variation in the fecal microbiota composition was observed in all septic critically ill patients. The diversity of the gut microbiota in both patients with severe and non-severe sepsis was significantly lower at ICU discharge than that at ICU admission with the extension of treatment time, leading to the expansion of clinically familiar pathogenic bacteria *Enterococcus faecium*.

### Supplementary Information

**Additional file 1: Supplemental Figure 1.** The microbial composition of fecal samples at the phylum level.

**Additional file 2: Supplemental Figure 2.** Serial changes in the ratio of Firmicutes to Bacteroidetes.

**Additional file 3: Supplemental Figure 3.** Association between Antibiotics and fecal microbiota composition at the phylum level in patients with sepsis.

## Data Availability

The datasets used and analyzed in the study are available from the corresponding author on reasonable request. The datasets generated during the current study are available in online repositories. The names of the repositories can be found below: https://www.ncbi.nlm.nih.gov/.

## Abbreviations

MODS: Multiple organ dysfunction syndrome
APACHE II: Acute Physiology and Chronic Health Evaluation II
SOFA: Sequential Organ Failure Assessment
OTUs: Operational Taxonomic Units
PCoA: Principal Coordinate Analysis
LEfSe: Linear discriminant analysis effect size
SD: Standard deviation
F/B: Firmicutes to Bacteroidetes
