# Supplementary figures and images for: Analysis of the dynamic changes in gut microbiota in patients with different severity in sepsis
Source: BMC Infect Dis. 2023 Sep 19;23:614. doi: 10.1186/s12879-023-08608-y (PMC10507951; doi:10.1186/s12879-023-08608-y)

**Supplemental Figure 1.The microbial composition of fecal samples at the phylum level.**

**
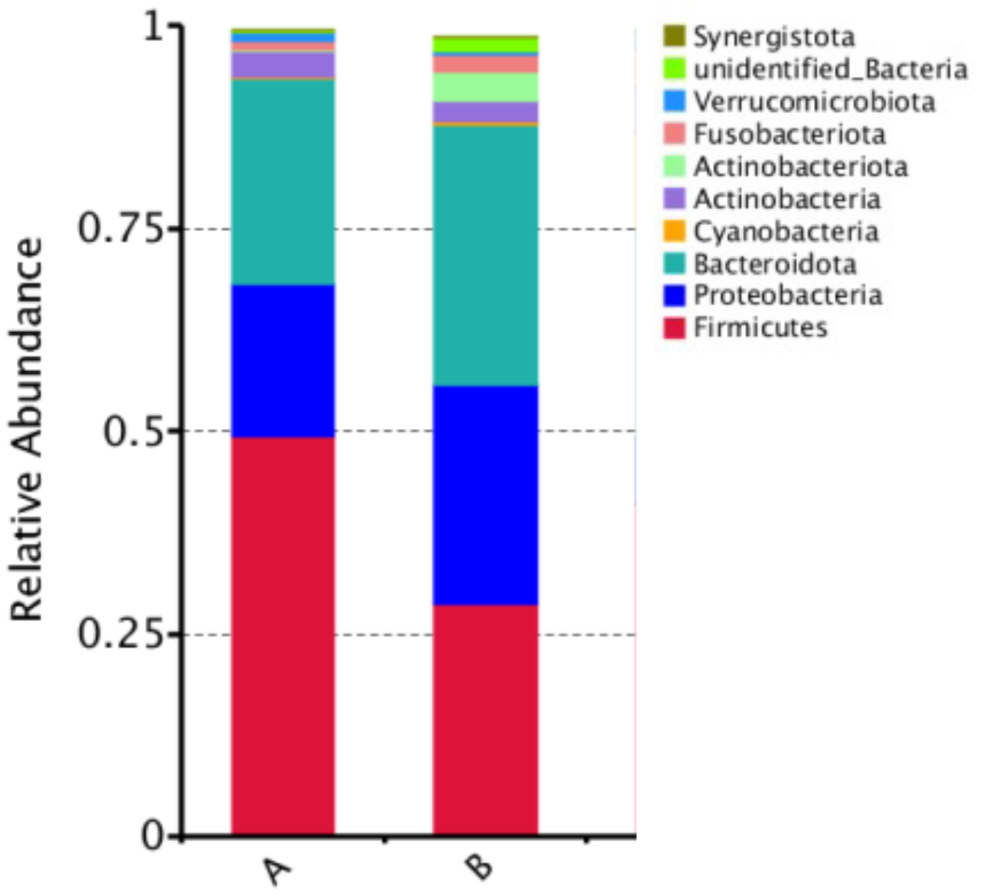
**

Supplement: Supplementary file 1 — Additional file 1: Supplemental Figure 1. The microbial composition of fecal samples at the phylum level. [file 12879_2023_8608_MOESM1_ESM.docx]

**Supplemental Figure** **2. Serial changes in the ratio of Firmicutes to Bacteroidetes.**

**
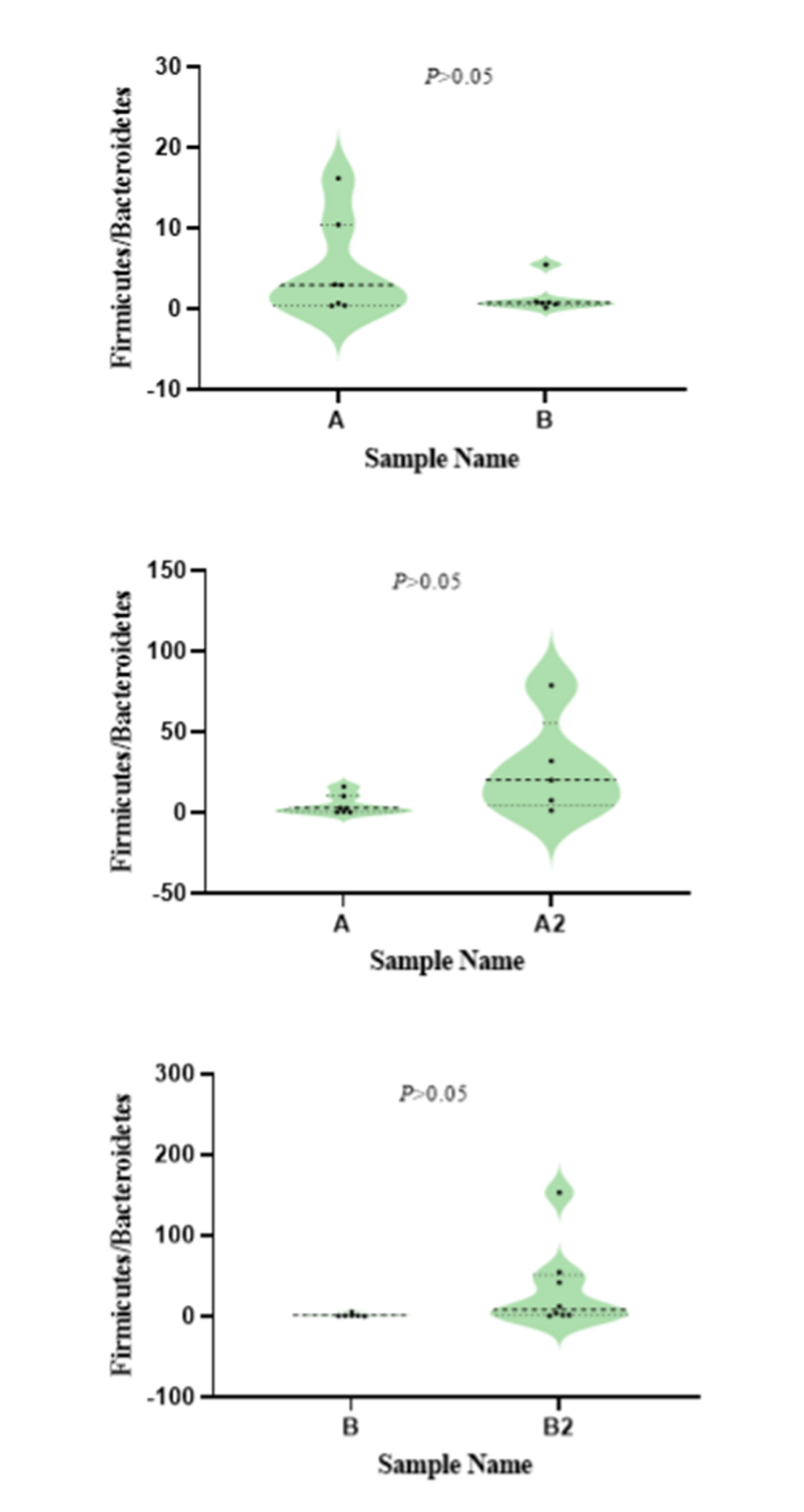
**

Supplement: Supplementary file 2 — Additional file 2: Supplemental Figure 2. Serial changes in the ratio of Firmicutes to Bacteroidetes. [file 12879_2023_8608_MOESM2_ESM.docx]
