# Supplementary material for: Analysis of the dynamic changes in gut microbiota in patients with different severity in sepsis
Source: BMC Infect Dis. 2023 Sep 19;23:614. doi: 10.1186/s12879-023-08608-y (PMC10507951; doi:10.1186/s12879-023-08608-y)
Supplement: Supplementary file 3 — Additional file 3: Supplemental Figure 3. Association between Antibiotics and fecal microbiota composition at the phylum level in patients with sepsis. [file 12879_2023_8608_MOESM3_ESM.docx]

**Supplemental Figure** **3. Association between Antibiotics and fecal microbiota composition at the phylum level in patients with sepsis.**

**
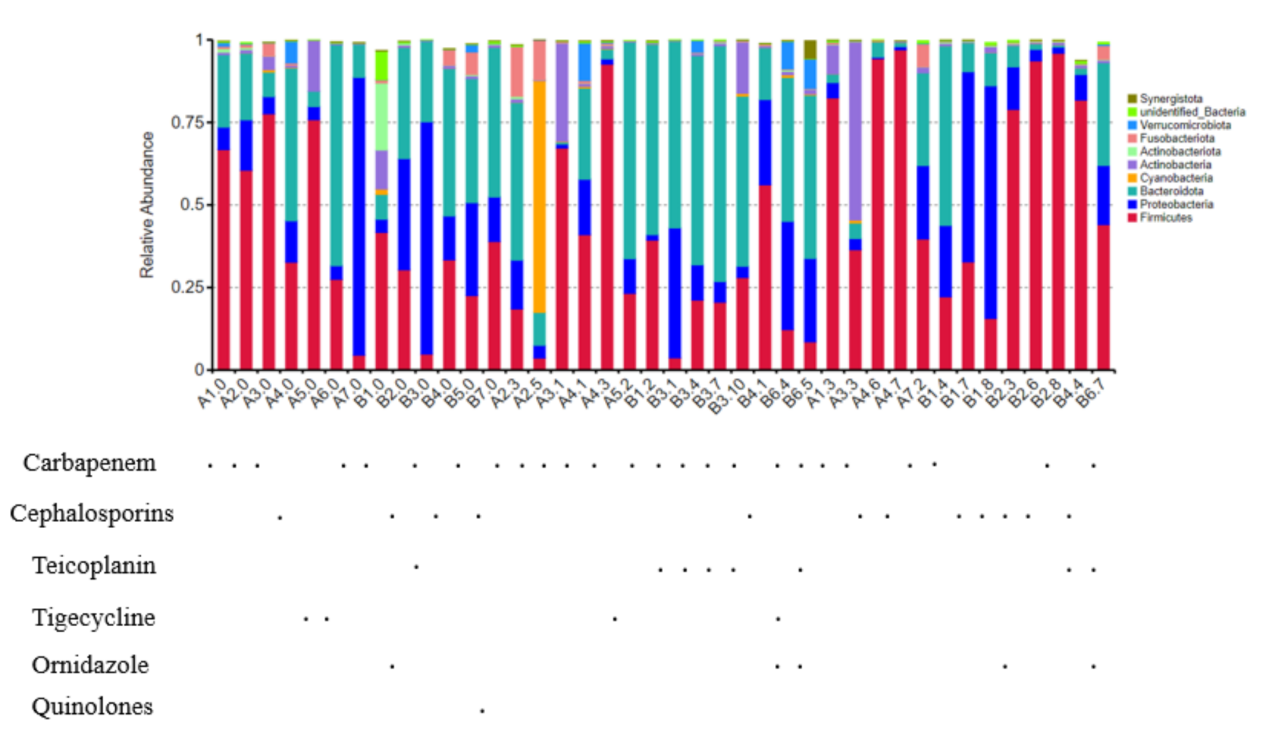
**
